# Size Reproducibility of Gadolinium Oxide Based Nanomagnetic Particles for Cellular Magnetic Resonance Imaging: Effects of Functionalization, Chemisorption and Reaction Conditions

**Published:** 2015

**Authors:** Sadjad Riyahi-Alam, Soheila Haghgoo, Ensieh Gorji, Nader Riyahi-Alam

**Affiliations:** a *School of Medicine, Tehran University of Medical Sciences (TUMS), Tehran, Iran*; b *Pharmaceutical Department, Food and Drug Laboratory Research Center, Food and Drug Organization (FDO), Ministry of Health, Tehran, Iran*; c *Medical Physics and Biomedical Engineering Department, School of Medicine, Tehran University of Medical Sciences (TUMS), Tehran, Iran*

**Keywords:** Nanomagnetic particles, Functionalization, Chemisorption, Cellular imaging, MRI contrast agents

## Abstract

We developed biofunctionalized nanoparticles with magnetic properties by immobilizing diethyleneglycol (DEG) on Gd_2_O_3_, and PEGilation of small particulate gadolinium oxide (SPGO) with two methoxy-polyethyleneglycol-silane (mPEG-Silane 550 and 2000 Da) using a new supervised polyol route, described recently. In conjunction to the previous study to achieve a high quality synthesis and increase in the product yield of nanoparticles; assessment of the effects of functionalization, chemisorption and altered reaction conditions, such as NaOH concentration, temperature, reaction time and their solubility, on size reproducibility were determined as the goals of this study. Moreover, the effects of centrifugation, filtration and dialysis of the solution on the nono magnetic particle size values and their stability against aggregation have been evaluated.

Optimization of reaction parameters led to strong coating of magnetic nanoparticles with the ligands which increases the reproducibility of particle size measurements. Furthermore, the ligand-coated nanoparticles showed enhanced colloidal stability as a result of the steric stabilization function of the ligands grafted on the surface of particles. The experiments showed that DEG and mPEG-silane (550 and 2000 Dalton) are chemisorbed on the particle surfaces of Gd_2_O_3_ and SPGO which led to particle sizes of 5.9 ± 0.13 nm, 51.3 ± 1.46 nm and 194.2 ± 22.1 nm, respectively. The small size of DEG-Gd_2_O_3_ is acceptably below the cutoff of 6nm, enabling easy diffusion through lymphatics and filtration from kidney, and thus provides a great deal of potential for further *in-vivo* and *in-vitro* application

## Introduction

Magnetic resonance imaging (MRI) is one of the various medical techniques in diagnosis of diseases. The signal of MRI is dependent on the T_1_ (spin–lattice relaxation time) and T_2_ (spin–spin relaxation time)(1). The relaxation times can be manipulated using magnetic compounds/chelates of gadolinium or iron oxides. The complexes of Gd^3+ ^or Mn^2+^ ions are paramagnetic contrast agents(CA) that causes brightening of MR images (positive contrast agents)(2). On the other hand, iron oxide particles (super paramagnetic contrast agents) darken the MR images and are known as negative contrast agents(3). Gd^+3^ based agents with seven unpaired f-electrons are widely opted for magnetic moment and applicability for different organs such as liver, spleen and lungs, where as, iron oxide is specific to liver. Despite the good magnetic properties, the free Gd^3+^ ion is extremely toxic. To reduce the toxicity, Gd^3+^ usually is complexed with strong organic chelators, *e.g*. diethylenetriaminepentaacetic acid (DTPA) which is used conventionally in daily MRI examinations (4). Due to the intrinsically low sensitivity of MRI, high local concentrations of the CA at the target site are required to generate higher image contrast. In addition, the MRI targeted CA should recognize targeted cells with high sensitivity materials such as nano magnetic particles (NMPs)(5). These particles should be biocompatible and have proper size and surface properties for optimum effects(size of the nanoparticles that are used in MRI are about 3 to 350 nm)(6). Therefore, physicochemical properties of nanoparticles will determine efficiency of nanoparticles, either polymeric or lipidic one. Nanoparticle surface modification with various coating materials is of utmost importance to prevent nanoparticle aggregation, decrease the toxicity, and increase the solubility and the bio compatibility(7,8). In recent years, extensive amount of experiments has been focused on the synthesis and surface modification of nanoparticles with high sensitivity characteristics(9,10,11). However, mechanisms of chemical synthesis, particle growth during formation, stability and reproducibility are still a challenge and require repetitive, accurate and cumbersome measurements. For this reason, it is quite important to develop methods in order to increase the product yield of nanomagnetic particles, control of the shell thickness, and elimination of the large size particles. Many of these coating materials, typically, involve some kind of polyethylene glycol (PEG) molecule or DEG(12,13). In the case of PEG, an intervening silane layer is often used for attaching the molecule to the nanoparticle. Furthermore, the PEG with various chain lengths has been used to alter the surface characteristics of magnetite.

The core-shell synthesis of magnetic nanoparticles will protect their surface from chemical reactions and the magnetic core from oxidation, causing hydrophobic effects and magnetic attractions, increasing cellular uptake rate, and possibility of various therapeutics attachment. Also, the biocompatibility of magnetic nanoparticles depends on the type of surface covering them, as well as, on their size. Likewise, coating of nanoparticles can increase relaxivity and half-life of the CA and protect them from aggregation. The total size of magnetic nanoparticles depends on the thickness of its coating such that nanoparticles coated with inorganic materials generally are smaller than 100 nm, where as polymer coating will result in larger particles above 100 nm(14). As a result, specific surface of the nanoparticles, and type of coating particle, will determine lipophilicity, surface charge and hydrophilicity of them. In magnetic liquids that are predominantly being prepared for biomedical applications, the surface charge which established by surface groups or by charge surface of the surrounding liquid medium results in a potential layer for physicochemical interactions(15).

We developed biofunctionalized nanoparticles with magnetic properties by coating of gadolinium oxide with diethyleneglycol (DEG), and PEGilation of small particulate gadolinium oxide (SPGO) with two different molecular weights of methoxy-poly ethyleneglycol-silane (mPEG-Silane 550 Da and mPEG-Silane 2000 Da)through a new supervised polyol route, introduced recently by this group(16). These types of NMPs are important in biosystems and expected to show higher contrast enhancement than that of commercially available CAs for MRI, such as Gd-DTPA. In a conjunction to the previous observations to achieve a high quality synthesis and increase in the product yield of NMPs, assessments of the effects of functionalization, chemisorption and altered reaction conditions on size reproducibility for increasing their stability against aggregation, were determined as the goals of this study. Also, a thorough description of the synthesis methods along with their chemical schemes was presented. Finally, after optimization of reaction parameters, we evaluated the magnetic properties the relaxometric measurements of the three contrast agents with different core-shells and molecular weights in comparison with the previous reports from PEGylation with larger molecular weights of 6000 Da and the conventional Gd-DTPA(11).

## Experimental

*Chemicals*

All the chemicals were in pure condition and used without further purification, and purchased as follows:Gd_2_O_3_, NaOH, DEG and SPGO nanoparticles (<40 nm and 99.999% pure) from Sigma-Aldrich (USA), mPEG-silane, MW550, from Nanocs, Inc.(MA, USA) and mPEG-silane, MW 2000, from Laysan Bio, Inc.(AL, USA).

*Physicochemical characterization*

The particle size of nano crystals was determined by Dynamic light scattering (DLS, Brookhaven Instruments-USA), and the measurements were repeated in different time intervals. Additionally, in the study of chemical reaction between DEG and Gd_2_O_3_, the sizes of nanoparticles were measured as functions of OH^-^ concentration of the solution, temperature and reflux time. Morphology of NMPs was examined by transmission electron microscopy (TEM, CM120 Model, Koninklijke, Philips Electronics, Netherlands). The chemical characteristics and reaction completeness of Gd_2_O_3_-DEG nanoparticles, prepared by the supervised polyol method before and after dialysis and centrifugation, and the chemical binding of mPEG-silane to the SPGO were investigated using Fourier transform infrared (FTIR) spectrophotometer (Tensor 27, Bruker Cor., Germany). FTIR spectra were obtained within the range of 4000-400 cm^-1^ at room temperature (26 ºC ± 1 ºC). Saturation of magnetization and superpar amagnetic characteristics of SPGO, Gd_2_O_3_-DEG and SPGO-mPEG-silane (550 and 2000 Dalton) were measured by vibrating sample magnetometer (VSM, 7400 model, Lakeshore Cryotronics Inc., OH, USA). At last, signal intensity and relaxivity measurements were performed using a GE 1.5-T MRI scanner (General Electric, WI, USA).

*Synthesis of Gd*_2_ *O*_3_ *-DEG nanocrystals*

**Figure 1:**
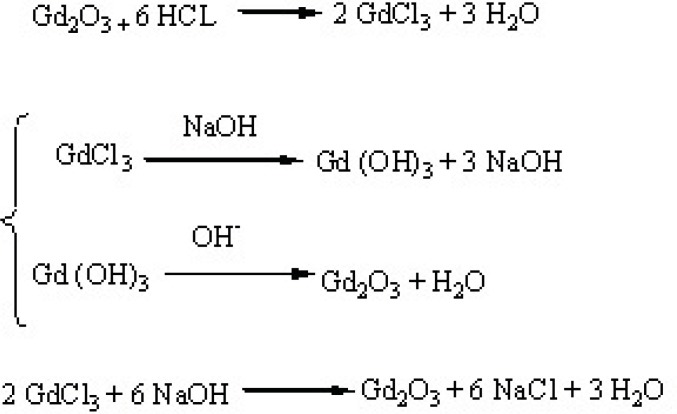
Reaction scheme showing the formation strategy of GdCl_3_·6H_2_O and Gd_2_O_3_

**Figure 2:**
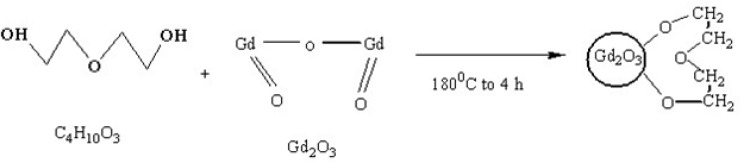
Reaction scheme showing the functionalization strategy when capping Gd_2_O_3_ nanoparticles with DEG, this reaction suggests a new configuration for DEG molecules, in which its oxygen binds two Gd atoms

NaOH Solutions were prepared by dissolving different amounts (0.3, 0.5 and 0.7 mM) of solid NaOH in 5 mL DEG and sonicated and/ or shook for 4 hours to get clear solution and were kept in proper condition for the subsequent steps. GdCl_3_·6H_2_O powder was prepared by dissolving 2 mmol Gd_2_O_3_ (not nano in size) in 1 mL HCl. On the experiment day, in a small reaction balloon, 0.9 mmol of GdCl_3_·6H_2_O was dissolved in 5 mL DEG by heating the mixture to 140 ^◦^C. After obtaining a clear solution, 5 mL of NaOH solution was added, and the temperature was raised to 180 ^◦^C for 4 h under reflux and magnetic stirring condition, leading to a dark yellow colloid. The solution was cooled, then the formed nano crystals were separated and filtered using centrifuge filtration at 2000 rpm(filters: polyethersulfone, 0.2 μm, Vivascience Sartorius, Hannover, Germany) for 30 min at 40 ^◦^C to remove agglomerations or large-size particles. Colloidal DEG–Gd_2_O_3_was dialyzed against deionized water in for 24 h using dialysis membrane (1000MW, Dialysis tubing benzoylated, Sigma-Aldrich, USA) to eliminate free Gd^3+^ ions and excess of DEG. These parts of work were not included in our previous study(17).The reaction scheme of capping Gd_2_O_3 _nanoparticles with DEG is shown in Figures 1 and 2.

*Synthesis of SPGO-mPEG nanocrystals*

**Figure 3:**
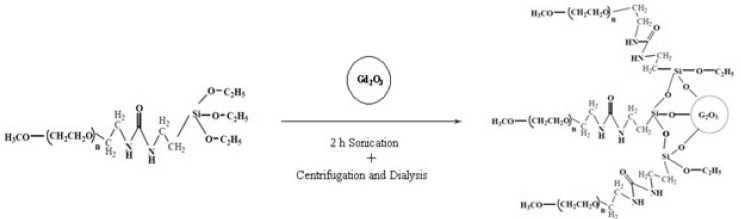
Reaction scheme showing the functionalization strategy when capping Gd_2_O_3_Nanoparticles with m-PEGSilane. This reaction shows mPEG-silane coated with SPGO by silane linker molecule.

To synthesize SPGO-mPEG nano crystals, 1 g of Gd_2_O_3_ (<40 nm) and 15 mgmL^−^^1 ^mPEG-silane(MW 550 or MW 2000) were mixed in 10 mL of deionized water and sonicated for 2 h at 40 ^◦^C. Agglomerations or large-size particles which may remain were eliminated in a similar fashion as above (*i.e*. centrifuged for 30 min, 2000 rpm at 40 ^◦^C). PEG–SPGO colloidal suspension was dialyzed in two separate steps: first using cylindrical dialysis membranes, (1000 MW), as described for DEG–Gd_2_O_3_ synthesis, free Gd^3+^ ions were eliminated. Second, cellulose membrane (12000 MW, Dialysis tubing cellulose membrane, Sigma-Aldrich, USA) was employed, in a separate tank for another 24 h, to remove free ligand plethora. Magnetic stirring was applied to increase the circulation in dialysis membrane, ensuring efficiency of the dialysis process. The capping reaction of Gd_2_O_3_ nanoparticle with mPEG-silane is shown in Figure 3.

## Results

*FTIR measurements*

**Figure 4:**
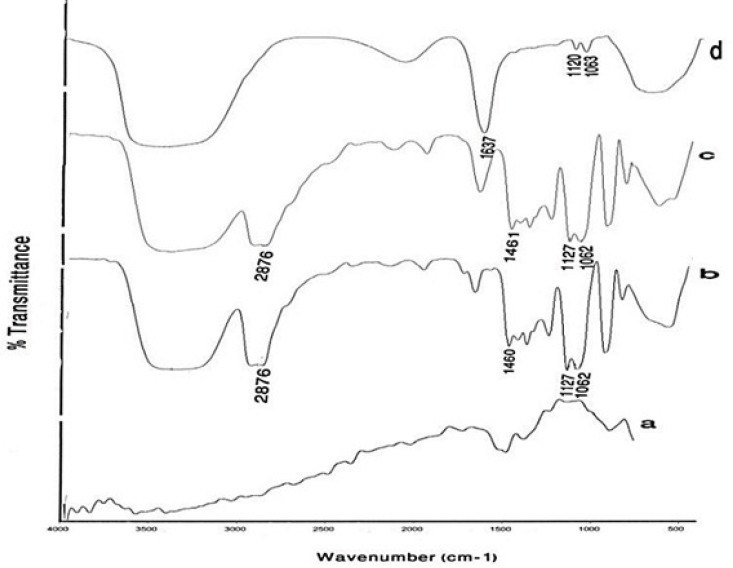
FTIR spectra of (a) a commercial Gd_2_O_3_ powder, (b) Pure DEG, (c) Gd_2_O_3 _nano crystals prepared by DEG coating without dialysis and centrifuge, (d) Gd_2_O_3_nanocrystals prepared in DEG after dialysis and centrifuge. Curves (c) and (d) depict the effects of the new supervised polyol synthesis route in chemical composition.

Figure 4 compares the FTIR spectrum of Gd_2_O_3 _powder, pure DEG, prepared Gd_2_O_3_-DEG nano crystals before and after centrifugation and dialysis process, in which characteristically different bands of ligands were detected. The bands in pure DEG (spectrum b) at 2876 and 1460 cm^−^^1^ correspond to symmetric stretching and bending of CH_2_. The band at 1127 cm^−^^1^ corresponds to C-O stretch, and the broad band of O-H stretch was observed in the 3100–3500 cm^−^^1 ^range. FTIR spectra b and c showed similar bands before the supervised polyol rout was applied. After centrifuge and dialysis, however, the FTIR spectrum corresponding to DEG, in the range of 1060-1130cm^−^^1^, diminished and shifted from 1127 to 1120 cm^−^^1^(C-O). Furthermore, the bands at1460 and 2876cm^−^^1^ (CH_2_) diminished as well (Figure 4d).

**Figure 5:**
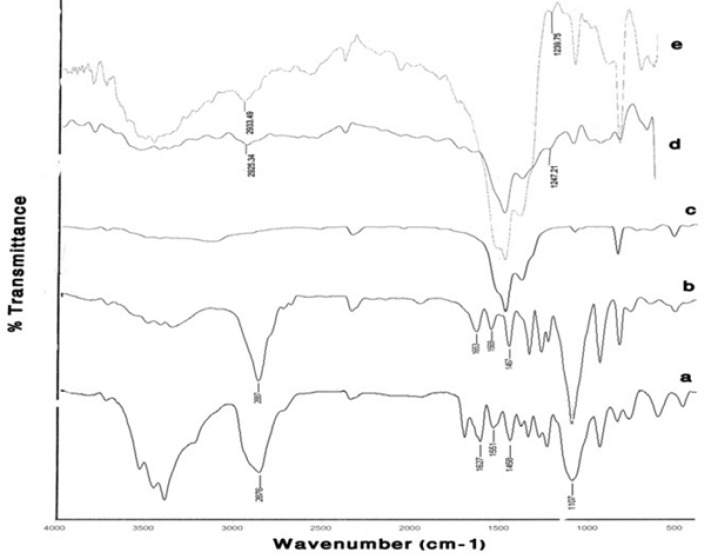
FT-IR spectra of (a) PEG-silane MW 550 powder, (b) PEG-silane MW 2000 powder, (c) a commercial SPGO powder, (d) PEG-silane MW 550 coated Gd_2_O_3 _nanoparticles and (e) PEG-silane MW 2000 coated Gd_2_O_3 _nanoparticles. Curves (a) and (d), and (c) and (e) show that for PEG-coated Gd_2_O_3_ nanoparticles, a silane linker molecule is used to couple the PEG to the nanoparticle

Figure 5 compares the results of FTIR spectroscopy for the two different mPEG-saline polymers, SPGO and the PEGylated SPGO nanoparticles (550 and 2000 Dalton). The spectrum of the PEG 550 Da (Figure 5a) showed characteristic peaks at 1284, 1627, 1107, 2876, 1458, 3100–3500 cm^-1^. Some of the strong absorptions of PEG are assigned for the -CH_2_CH_2_- symmetric stretching and bending around 2876 and 1458 cm^-1^(18,19)which demonstrate the presence of saturated carbons -(CH_2_CH_2_)_n_-.The Peak at 1284 cm^-1^ corresponds to Si–C stretching vibration. The bands at 1627 and 1107 correspond to C=O stretching vibration and C-O ether stretching vibration, respectively. The band at 1551 cm^-1^ corresponds to –NH bending vibration in the amide located between the silane and the PEG. Noticeably broad bands in the 3100 and 3600 cm^-1^ region indicate exchangeable protons in N-H. Spectra d and e (Figure 5)belong to SPGO-PEG nanoparticles, whereas spectrum c (Figure 5) belongs to SPGO before adding PEG. As can be seen, pure SPGO possesses characteristic peaks at 850 and 1500 cm^-1^. In addition, two shifts of PEG-silane 550 Da bands’ peaks from 1284 to 1247.21 and from 2876 to 2925 cm^-1^(Figure 5d) were exhibited. It should be noted that the PEG coated SPGO particles were dialyzed before measurements, to remove the excess of PEG polymers (*i.e*. PEG polymers that were physically absorbed onto the surface of the particles), and the observed signals, thus only belong to the PEG polymers that are chemically attached. There are only small differences in the FTIR spectrum of SPGO-mPEG-silane 2000 Da compared to that of SPGO-mPEG-silane MW 550 (Figure 5e).

*Particle size measurements*

**Figure 6:**
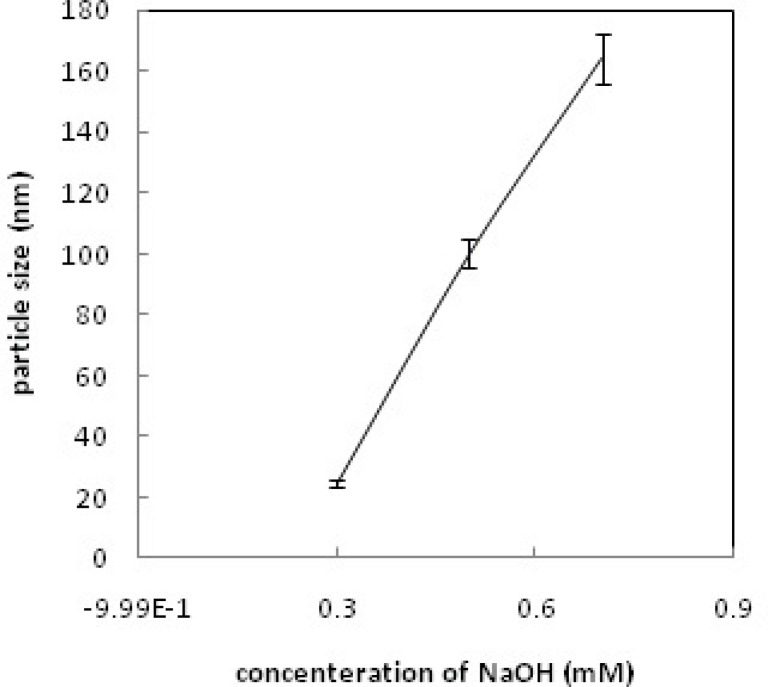
Particle size vs. OH^-^ concentration.

**Figure 7:**
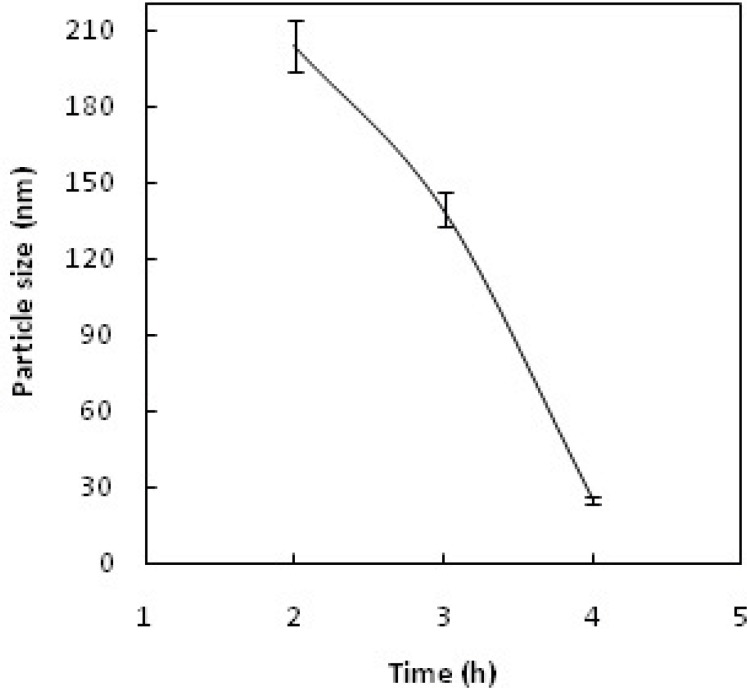
Particle size vs. reaction reflux time.

**Figure 8:**
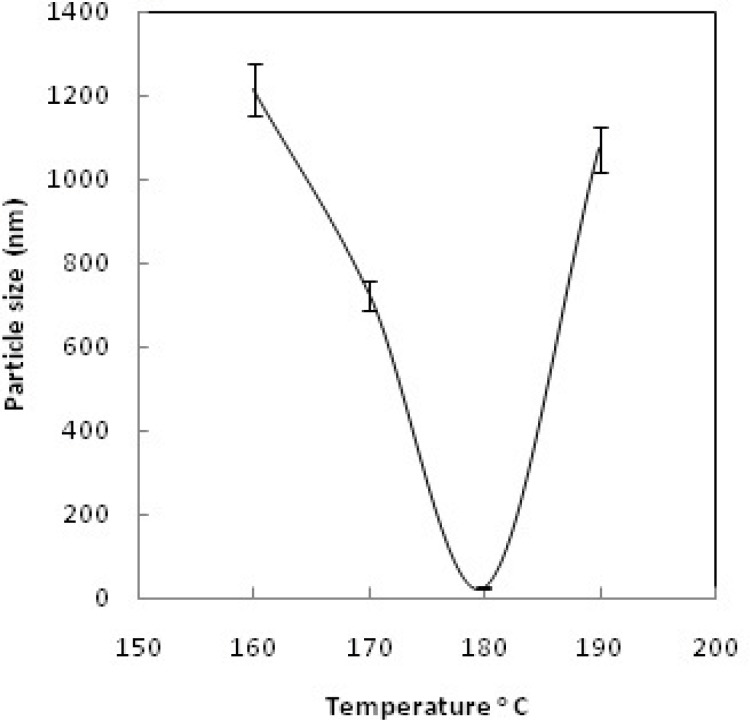
Particle size vs. reaction temperature.

Dynamic light scattering (DLS) was used for estimating the hydrodynamic radius of the nanoparticles. Figure 6 shows relationship between particle size and concentration of OH^-^; increasing the concentration of OH^- ^increases the particle size and leads to rapid precipitation of nanoparticles. Figure 7 shows the recorded Gd_2_O_3_-DEG nano crystal sizes as a function of refluxing time in the reaction. The results indicated that increasing the reaction time causes decrease in the size of the nanoparticles(*i.e.* the smallest particle sizes were obtained after 4 h reflux). Figure 8 shows the size of the particles relative to the reaction temperature (NaOH 0.3 mM, 4 h). The smallest size was obtained at 180 ºC, while increasing the temperature from 180 to 190 ºC caused aggregation of the particles. nanoparticles in optimization process reached nearly 20 nm in size; still they needed to undergo filtration and dialysis. Thereby, in the optimum reaction conditions, and after filtration and dialysis, the small proper particle size of 5.9 ± 0.13 nm (respective pdI of 0.387) was obtained (Table 1), compared to the larger size in our previous study(14).

Note that the increase in the concentration is associated with increase in particle size.

According to the curve, size is inversely related to reflux time

In 180 ºC, the smallest particle size was obtained.

**Figure 9:**
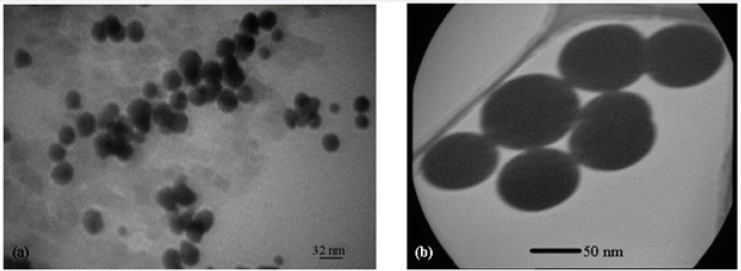
(a) and (b) TEM images of Gd_2_O_3_-DEG nanocrystals, showing high resolution images of well uniformed and separated gadolinium nanoparticles after coating by DEG chelates.

**Figure 10:**
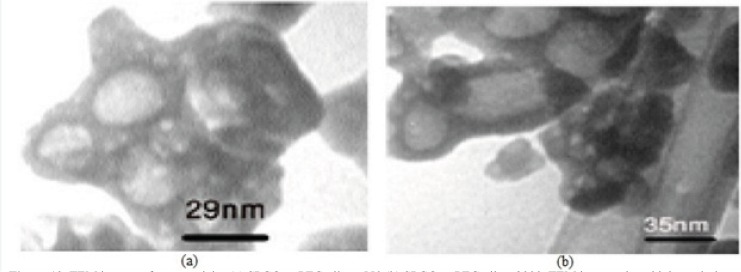
TEM images of nanoparticles (a**) **SPGO–mPEG-silane 550 (b) SPGO–mPEG-silane2000. TEM images show high resolution images of separated gadolinium nanoparticles after coating with PEG chelates. These particles were agglomerated and couldn’t show images as sharp as that of DEG coated particles

Figure 9 shows the TEM images of Gd_2_O_3_-DEG nanocrystals, used for hydrostatic size measurements. Gd nanomagnetic particles are clearly formed in uniform spherical or ellipsoidal shapes and visualized separately in nano scaled grains. These findings show that main nucleus (Gd_2_O_3 _core) is coated by DEG molecules through a strong interaction between DEG with the Gd_2_O_3_ nanoparticle surface. Figure 10 shows TEM images of two other PEGylated nanoparticles, which in contrast to Gd_2_O_3_-DEG nanoparticles, PEG coated NPMs were not visualized as evidently due to agglomeration and their large molecular weights.

Table 1 shows the size and polydispersity index (PdI) measurements using DLS for Gd_2_O_3_-DEG and SPGO-PEG 550 and 2000 Dalton nanoparticles. Hydrodynamic radiuses of filtered and dialyzed nanoparticles were 51.3 ± 1.46 nm and 194.2 ± 22.1 nm, with PdI of 0.350 and 0.225 for PEG 550 and PEG 2000 coated particles, respectively.

**Table 1:** DLS size and PdI measurements for the three nanoparticles

**PdI**	**Hydrodynamic diameter(nm)**	**Nanoparticle**
0.387	5.9 ± 0.13	DEG-Gd_2_O_3_
0.350	51.3 ± 1.46	SPGO-mPEG-silane550
0.225	194.2 ± 22	SPGO-mPEG-silane2000

The results show a direct relationship between size and molecular weight.

*Analysis of magnetic properties*

**Figure 11:**
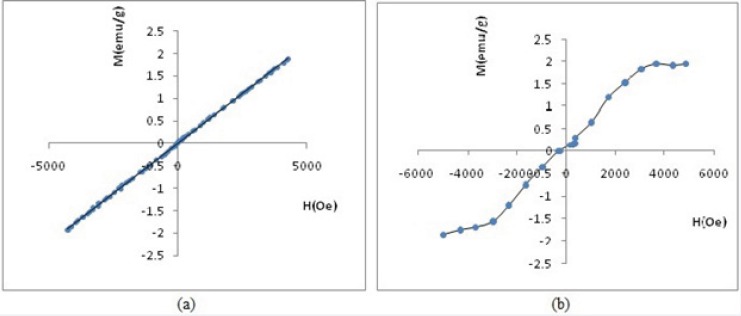
(a) Hysteresis loop by VSM of SPGO particles (b) Hysteresis loop by VSM of Gd_2_O_3_-DEG

**Figure 12:**
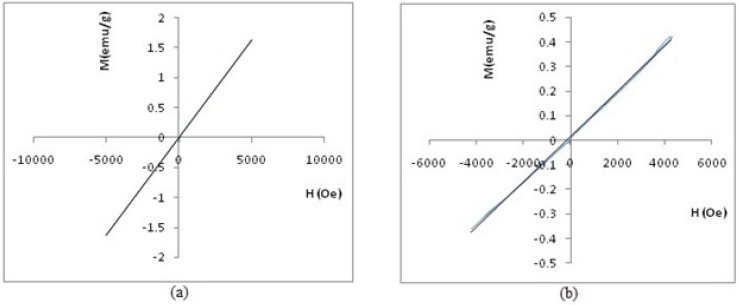
Hysteresis loop by VSM of **(**a) Gd_2_O_3_-PEG 550 Da nanoparticles and (b**)** Gd_2_O_3_-PEG 2000 Da nanoparticles.

Measurements of magnetic properties were done using VSM and at room temperature. Figures 11 and 12 demonstrate the relationship between relative magnetization curves and applied field. Removing the applied magnetic field will not lead to coercivity and remanence in paramagnetic, diamagnetic and super para magnetic materials. Paramagnetic materials also have a linear relationship between their magnetization (M) and applied field (H) with positive slope. Figure 11a vividly shows paramagnetic properties of SPGO particles. Where, Gd_2_O_3_-DEG nanoparticles exhibited S shape (sigmoidal) Magnetization curve of super paramagnetic materials in Figure 11b.

nanoparticle. The differences between SPGO and Gd_2_O_3_-DEG VSM curves, due to the effect of covering, can be seen evidently.

Figure 12 shows the magnetometry of PEGylated nanoparticles. A linear relationship is apparent between magnetization (M) and applied field in this figure, thus, inferring that these two PEGylated nanoparticles are paramagnetic materials. Please note that the susceptibility (as slope of curve) for SPGO-mPEG-silane2000 is less than that of SPGO-mPEG-silane550 (κ_2000_=9.20×10^-5 ^<κ_550_=3.28×10^-4^).

*Maximum signal intensities in different concentrations and relaxivity measurements*

**Figure 13:**
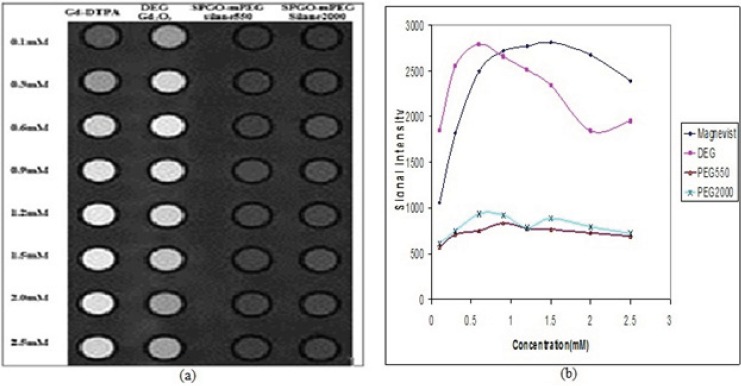
Signal intensity **(**a) Images and (b) curves for Gd-DTPA Gd_2_O_3-_DEG , SPGO–mPEG-silane550, and SPGO–mPEG-silane2000 in different concentrations, using standard spin echo imaging with TR/TE=600/15ms. The quantitative variations of signal intensities in (b**)** is in complete agreement with the image visualization in (a), for *in-vitro* dilutions of the three nanoparticles

Signal intensity Images and curves for Gd-DTPA, Gd_2_O_3_-DEG, SPGO-mPEG-silane550 and SPGOm-PEG-silane 2000 using standard spin echo imaging with TR/TE=600/15ms has been presented in Figure 13. The results of quantitative variation of signal intensities in Figure 13(b) are in complete accordance with the image visualization in Figure 13(a) for *in-vitro* dilutions of the four materials. Concentrations of 0.6, 0.6 and 0.9 mM corresponded to the maximum signal intensities for Gd_2_O_3_-DEG, SPGO-mPEG-silane550 and SPGO-mPEG-silane2000, respectively. Table 2 shows the r_1_ and r_2_ as the slope of R_1_ and R_2_ relaxation rates versus concentration values for Gd-DTPA, and the three nanoparticles in water.

**Table 2:** Results of relaxometry for three nanoparticle contrast agents and Gd-DTPA

r_2_/r_1_( relaxivity ratios)	*r*_2_ (mM^−1^ s^−1^)	*r*_1 _(mM^−1^ s^−1^)	Nanoparticle
1.13	5.14	4.55	Gd-DTPA
0.89	11.81	13.31	DEG-Gd_2_O_3_
37.62	26.34	0.70	SPGO-mPEG-silane550
33.72	33.72	1.00	SPGO-mPEG-silane2000

Please note that longitudinal relaxivity (r1) of Gd-DTPA and PEGylated nanoparticles (SPGO-mPEGsilane550

and 2000) were smaller than that of Gd_2_O_3_-DEG

## Discussion

In this study, Gd_2_O_3_ nanoparticles with three different core-shells (DEG, m-PEG Silane 550 and 2000 Dalton) were synthesized, functionalized and dialyzed for further *in-vitro* and *in-vivo* applications in biological systems (Figures 1-3). This was done through the newly described supervised polyol rout, in our previous experiment(16). Using this method, we were able to obtain substantially small size of about 6 nm for DEG coated Gd_2_O_3_. FTIR results for Gd_2_O_3 _powder andGd_2_O_3_-DEG nanoparticles in Figure 4, showed no significant differences between b and c spectra, accounting for over abundance of DEG molecules. After purification of the DEG coated Gd_2_O_3 _nanoparticles with centrifuge and dialysis, unreacted DEG has been removed. Consequently, in the FTIR spectrum of Gd_2_O_3_-DEG nanoparticles, diminishing and shifting of DEG band peaks to lower frequencies were observed; especially, for position of CH_2 _and C-O stretching bands of DEG which could be due to surface interaction and chemisorptions with Gd_2_O_3 _particles. The shift in C-O peak from 1127 to 1120 cm^−^^1^ can suggest a new configuration for DEG molecules, in which its oxygen binds with two Gd atoms (Figure 5). This has also been reported by Pedersen (20). In SPGO-mPEG-silane nanoparticles (550 and 2000 MW), Gd_2_O_3 _nucleates grow to form Gd_2_O_3 _nano crystals, and are subsequently capped and stabilized by mPEG-silane.A silane molecule can act as a linker to help chemisorbtion of the PEG polymer on the nanoparticle surface (Figure 3) which is in great agreement with the FTIR results (Figure 5). The shifts of the characteristic peaks of the PEG-silane 550 Da, from 1284 to 1247.21 and from 2876 to 2925 cm^-1^(Figure 5, Spectrum d) are strong evidence that PEG is bonded to the surface of SPGO through a reaction of PEG silane 550 Da with the nanoparticles, also reported by Wu (21). Spectrum e, Figure 5, showed very similar results for FTIR spectrum of PEGylated SPGO particles with mPEG-silane 2000 Da to that of SPGO-mPEG-silane MW 550. The small differences observed between them are due to the size effect or molecular weight.

Different sizes of Gd nanoparticles, in the range of 20 nm, coated with DEG were obtained through investigating the alteration of reaction conditions. High yield of DEG coating, however, may be achieved by adjusting NaOH concentration, temperature and reaction time of solution to 0.3 mM, 180ºC and 4 h, respectively. Considering the thermodynamical instability of products in a prolonged period of time due to aggregation, fusion and precipitation in stacks of infinite; size measurements have been repeatedly performed in a month, year and even longer time intervals to assure the physical characteristic stability of NMPs. Obtained poly disperesity Index (PdI) by DLS, indicative of hydrodynamic diameter distributions(Table 1), and also morphology and hydrostatic diameter distributions, presented by TEM (Figure 9-10), were employed as the stability measures. The results of these serial measurements, however, revealed no significant changes of the Gd_2_O_3_-DEG/PEG sizes, as well as, repeated relaxometry measurements with no significant differences (Table 2), imply the chemical stability of the products during the experimental period. And these findings are evidence of absence of degradation and oxidation during the imaging protocols. Eventually, filtration and dialysis of particles yielded in optimum reaction condition, after which we achieved above mentioned hydrodynamic distribution of 5.9 nm.

In order to evaluate the influence of the chain lengths of coating agents, two PEG molecules with different molecular weights (PEG550–OCH_3_, PEG2000–OCH_3_) were selected. These PEG chains carry a reactive group at one end for grafting onto the surface of the particles, and a methoxy group at the other extremity (PEG550–OCH_3_, PEG2000–OCH_3_) which influences the colloidal stability and biodistribution. Thus, they have similar terminal groups but differ in their chain lengths. The effect of the molecular weight of PEG on the colloidal stability and size of nanoparticles was also investigated. Our findings in Table 1 showed that molecular weight can affect particle size. The difference between size distributions of Gd-nanoparticles coated with mPEG-silane 550 and 2000 polymers were observed by the light scattering method(22). This difference in sizes for PEG 550 and 2000 nanoparticles molecules can be deduced from the different steric stabilization effects of these two polymers on nanoparticles (Figure 10). PEG-2000 extends into the medium in the form of a thicker layer on the particle surface as a result of the longer side chains, compared to PEG 550. These larger side chains, causing steric instability in the media, led PEG 2000 not to be as effective a steric stabilizer as PEG 550. Thus they aggregated, and as a result larger sizes were observed.

Several studies have been done related to the effect of particle size on magnetic properties or relaxivities. Some of these investigations have also showed that the relaxation ratios increase with larger sizes of nanoparticles(23,24,25). In this endeavor, after obtaining the NMPs particles with proper size, by altering reaction conditions, magnetic properties and relaxivities were investigated. The relaxometric measurements showed significant magnetic properties for Gd_2_O_3_-DEG compared to the conventional Gd-DTPA with relaxivity ratios of 0.89 and 1.13 (Table 2)(26), that in part is due to the small size of the nanoparticles. Figure 11a showed that Gd_2_O_3_-DEG reached its maximum signal intensity at a concentration close to the daily clinical concentration of Gd-DTPA (*i.e*. 0.1 mM). Also for SPGO-mPEG-silane (550 and 2000 Dalton) with lower relaxivity ratios(37.62 and 33.72 respectively)compared to that of previous reports of PEGylation with larger molecular weights(6000 Dalton)(11), showed more promising results as a negative contrast agent.

At last but not least, surface properties and particle size are crucial factors for cell internalization through the plasma membrane. Studies have shown that nanoparticles smaller than 50 nm of size or with lipophilic polymer coatings diffuse across cell membranes easily(27). The size of approximately 6nm,which we acquired, is exceptionally important in that the particles up to 6nm are easily diffused from lymphatics and typically filtered through the glumerolar capillary(28,29). Another advantage of surface modification is increased circulation time of nanoparticles in blood stream, by avoiding agglomeration and protein adsorption. Thus, they can reach target cells without being phagocytosed (30)All these characteristics can potentially hold for Gd_2_O_3_-DEG, as well.

## Conclusion

Optimization of reaction parameters leads to strong coating of nanoparticles with the ligands which in turn increases the reproducibility of particle size measurements. Besides, the ligand-coated nanoparticles can show the enhanced colloidal stability as a result of the strict stabilization function of the ligands grafted on the surface of particles. Thus, the relaxometric measurements will lead to significant positive magnetic properties for Gd_2_O_3_-DEG compared to conventional Gd-DTPA and also better results as a negative contrast agent for SPGO-mPEG-silane (550 and 2000 Dalton) with lower r_2_/r_1 _(relaxometry ratio)compared to higher MW polymers. Moreover, Optimal clearance resulting in less toxicity, lymphatic diffusion and increased circulation, which all can be attributed to Gd_2_O_3_-DEG nanoparticles, altogether hold promise for further investigation of these nanomagnetic particles *in*-*vitro* and *in*-*vivo*, for cellular and molecular imaging for cancer and other diagnostic purposes.

## References

[1] Haacke EM (1999). Magnetic Resonance Imaging: Physical Principles and Sequence Design.

[2] Aime S, Fasano M, Terreno E (1998). Lanthanide(III) chelates for NMR biomedical applications. Chem. Soc. Rev.

[3] Bulte JW, Kraitchman DL (2004). Iron oxide MR contrast agents for molecular and cellular imaging. NMR Biomed.

[4] Laurent S, Elst LV, Muller RN (2006). Comparative study of the physicochemical properties of six clinical low molecular weight gadolinium contrast agents. Contrast Media Mol. Imaging.

[5] Artemov D (2003). Molecular magnetic resonance imaging with targeted contrast agents. J. Cell Biochem..

[6] Waters E, Wickline S (2008). Contrast agents for MRI. Basic Res. Cardiol..

[7] Soderlind F, Pedersen H, Petoral RM Jr, Kall PO, Uvdal K (2005). Synthesis and characterisation of Gd2O3 nanocrystals functionalised by organic acids. J. Colloid Interface Sci..

[8] Derakhshandeh K, Hochhaus G, Dadashzadeh S (2011). In-vitro Cellular Uptake and Transport Study of 9-Nitrocamptothecin PLGA Nanoparticles Across Caco-2 Cell Monolayer Model. Iran. J. Pharm. Res..

[9] Engstrom M, Klasson A, Pedersen H, Vahlberg C, Kall PO, Uvdal K (2006). High proton relaxivity for gadolinium oxide nanoparticles. MAGMA.

[10] Klasson A, Ahren M, Hellqvist E, Soderlind F, Rosen A, Kall PO, Uvdal K, Engstrom M (2008). Positive MRI contrast enhancement in THP-1 cells with Gd2O3 nanoparticles. Contrast Media Mol. Imaging.

[11] Nelson JA, Bennett LH, Wagner MJ (2002). Solution synthesis of gadolinium nanoparticles. J. Am. Chem. Soc..

[12] Xu H, Yan F, Monson EE, Kopelman R (2003). Room-temperature preparation and characterization of poly (ethylene glycol)-coated silica nanoparticles for biomedical applications. J. Biomed. Mater Res. A.

[13] Sathish Kumar K, Jaikumar V (2011). Gold and iron oxide nanoparticle-based ethylcellulose nanocapsules for Cisplatin drug delivery. Iran. J. Pharm. Res..

[14] Na HB, Song IC, Hyeon T (2009). Inorganic Nanoparticles for MRI Contrast Agents. Adv. Materials.

[15] Liu Y, Chen Z, N Z (2011). Novel nanovectors as liver targeting MRI contrast agents. J. Chinese Pharm. Sci..

[16] Azizian G, Riyahi-Alam N, Haghgoo S, Moghimi HR, Zohdiaghdam R, Rafiei B, Gorji E (2012). Synthesis route and three different core-shell impacts on magnetic characterization of gadolinium oxide-based nanoparticles as new contrast agents for molecular magnetic resonance imaging. Nanoscale Res. Lett..

[17] Riyahi-Alam N, Behrouzkia Z, Seifalian A, Haghgoo Jahromi S (2010). Properties evaluation of a new MRI contrast agent based on Gd-loaded nanoparticles. Biol. Trace Elem. Res..

[18] Alcantar NA, Aydil ES, Israelachvili JN (2000). Polyethylene glycol-coated biocompatible surfaces. J. Biomed. Mater Res..

[19] Matsuura H, Miyazawa T (1969). Vibrational analysis of molten poly(ethylene glycol) Part A-2: Polymer Physics. J. Polymer Sci.

[20] Pedersen H, Söderlind F, Petoral Jr RM, Uvdal K, Käll P-O, Ojamäe L (2005). Surface interactions between Y2O3 nanocrystals and organic molecules—an experimental and quantum-chemical study. Surface Sci.

[21] Wu Y, Zuo F, Zheng Z, Ding X, Peng Y (2009). A Novel Approach to Molecular Recognition Surface of Magnetic Nanoparticles Based on Host-Guest Effect. Nanoscale Res. Lett.

[22] Bruce J, Berne and Pecora R (2000). Dynamic Light Scattering: With Applications to Chemistry, Biology, and Physics.. Courier Dover Publications.

[23] Faucher L, Gossuin Y, Hocq A, Fortin M-A (2011). Impact of agglomeration on the relaxometric properties of paramagnetic ultra-small gadolinium oxide nanoparticles. Nanotechnol.

[24] Fortin M-A, Jr RMP, Söderlind F, Klasson A, Engström M, Veres T, Käll P-O, Uvdal K (2007). Polyethylene glycol-covered ultra-small Gd 2 O 3 nanoparticles for positive contrast at 1. 5 T magnetic resonance clinical scanning.. Nanotechnol.

[25] Li Y, Pei Y, Zhang X, Gu Z, Zhou Z, Yuan W, Zhou J, Zhu J, Gao X (2001). PEGylated PLGA nanoparticles as protein carriers: synthesis, preparation and biodistribution in rats. J. Control Release.

[26] Azizian G, Riyahi-Alam N, Haghgoo S, Saffari M, Zohdiaghdam R, Gorji E (2013). Safety assessment of nanoparamagnetic contrast agents with different coatings for molecular MRI. Materials Science-Poland.

[27] Sun C, Lee JS, Zhang M (2008). Magnetic nanoparticles in MR imaging and drug delivery. Adv. Drug Deliv. Rev.

[28] Barrett T, Choyke PL, Kobayashi H (2006). Imaging of the lymphatic system: new horizons. Contrast Media Mol. Imaging.

[29] Longmire M, Choyke PL, Kobayashi H (2008). Clearance properties of nano-sized particles and molecules as imaging agents: considerations and caveats. Nanomed (Lond).

[30] Zhang Y, Kohler N, Zhang M (2002). Surface modification of superparamagnetic magnetite nanoparticles and their intracellular uptake. Biomaterials.

